# EUV and Hard X-ray Hartmann Wavefront Sensing for Optical Metrology, Alignment and Phase Imaging

**DOI:** 10.3390/s21030874

**Published:** 2021-01-28

**Authors:** Ombeline de La Rochefoucauld, Guillaume Dovillaire, Fabrice Harms, Mourad Idir, Lei Huang, Xavier Levecq, Martin Piponnier, Philippe Zeitoun

**Affiliations:** 1 Imagine Optic, 18 rue Charles de Gaulle, 91400 Orsay, France; gdovillaire@imagine-optic.com (G.D.); fharms@imagine-optic.com (F.H.); xlevecq@imagine-optic.com (X.L.); mpiponnier@imagine-optic.com (M.P.); 2 Brookhaven National Laboratory, 50 Rutherford Drive, Upton, NY 11973, USA; midir@bnl.gov (M.I.); lhuang@bnl.gov (L.H.); 3 Laboratoire d’Optique Appliquée, CNRS, ENSTA Paris, Ecole Polytechnique IP Paris, 91120 Palaiseau, France; philippe.zeitoun@ensta-paris.fr

**Keywords:** EUV wavefront sensor, X-ray wavefront sensor, Hartmann sensor, phase imaging, metrology, X-ray sources

## Abstract

For more than 15 years, Imagine Optic have developed Extreme Ultra Violet (EUV) and X-ray Hartmann wavefront sensors for metrology and imaging applications. These sensors are compatible with a wide range of X-ray sources: from synchrotrons, Free Electron Lasers, laser-driven betatron and plasma-based EUV lasers to High Harmonic Generation. In this paper, we first describe the principle of a Hartmann sensor and give some key parameters to design a high-performance sensor. We also present different applications from metrology (for manual or automatic alignment of optics), to soft X-ray source optimization and X-ray imaging.

## 1. Introduction

The emergence of ultrafast Extreme Ultra Violet (EUV) to X-ray sources, namely Free Electron Laser, High Harmonic Generation, laser-driven betatron and Compton, and the emergence of Diffraction Limited Storage Ring has opened the door to new, exciting experiments in physical, chemical and biological sciences. Many of these experiments require an optimum or at least a properly defined beam wavefront (WF). Several X-rays WF sensing techniques have been proposed in the past: grating-based interferometry [1], speckle tracking [2,3], ptychography [4,5,6] and imaged-based wavefront sensing combined with machine learning [7]. Among these techniques, Hartmann WF sensing provides several advantages, such as achromaticity, very large dynamic range, real-time wavefront display and ease of use [8,9,10,11]. Derivative methods of the Hartmann approach were developed by Freisem and collaborators [12] by replacing the Hartmann plate by a transmission grating allowing them to simultaneously detect harmonic wavefronts and resolve the spectra.

Even if the Hartmann method relies on a quite simple approach, the demanding accuracy underlying the very short wavelengths as well as the technological constraints linked to this spectral domain make the manufacturing of a Hartmann sensor at these energies very difficult. Since 2003, Imagine Optic has been developing EUV to X-rays Hartmann WF sensors. In this paper, we describe the principle of such WF sensors. We firstly focus on the calibration step required to enable absolute WF sensing, and then present a broad range of applications: from beamline and optical setup optimization, understanding of some physics phenomena, characterization of EUV to X-ray sources and finally some phase imaging experiment. Only results with the Hartmann sensors developed by Imagine Optic are presented.

## 2. Hartmann Wavefront Sensors

### 2.1. Principle of A Hartmann Wavefront Sensor

**Figure 1:**
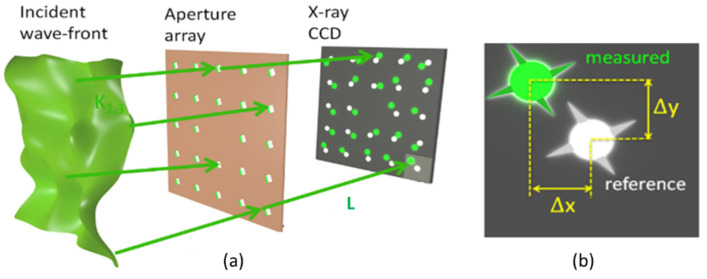
(**a**) Schematic representation of the incoming aberrated wave (in green), going through the Hartmann plate and reaching the detector. (**b**) The white dots correspond to the diffraction spots when a perfect beam reaches the detector, while the green dots correspond to an unknown beam. Courtesy of Dr. Li Lu.

A Hartmann WF sensor is mainly composed of a grid of holes placed at a certain distance from a 2D detector, as illustrated in Figure 1. The principle is quite simple: the beam reaching the matrix of holes is divided into beamlets locally sampling the wavefront of the incoming beam. At this step, a major assumption is made that assumes the WF to be flat over the aperture of the hole. The diffraction from each hole creates a spot on the detector. Their positions are directly proportional to the local derivative of the WF (i.e., the local wave vector direction). A perfect knowledge of the reference positions of the spots—obtained from a perfect WF—and of the geometrical parameters of the system (distances, position of the holes etc.) allows us to perform an absolute determination of the incident WF.

When an aberrated beam reaches the detector, the spots are locally shifted by Δ*x* in the X direction (Δ*y* in the Y direction) as compared to the reference positions (green versus white dots, Figure 1). The well-established centroid method was used to measure accurately the relative displacements within each subaperture of the grid. Let us consider a distance *L* between the mask and the detector. From the measured Δ*x* and Δ*y*, the local slopes of the incoming WF in both directions ($S_{i,j}^{x},\;S_{i,j}^{y})$ can be calculated at the spot (*i,j*) on the detector:

(1) $$S_{i,j}^{x}=tan\left(\frac{\Delta x_{i,j}}{L}\right)\approx \frac{\Delta x_{i,j}}{L}=\frac{\lambda }{2\pi }\frac{d\varphi \left(x_{i,j},y_{i,j}\right)}{dx}$$

(2) $$S_{i,j}^{y}=tan\left(\frac{\Delta y_{i,j}}{L}\right)\approx \frac{\Delta y_{i,j}}{L}=\frac{\lambda }{2\pi }\frac{d\varphi \left(x_{i,j},y_{i,j}\right)}{dy}$$

An integration of the slope maps $S_{i,j}^{x}$ and $S_{i,j}^{y}$ along X and Y directions gives the phase map $\varphi \left(x_{i,j},y_{i,j}\right)$. Wavefront aberrations can be described by Zernike or Legendre polynomials. More details about the data analysis and the wavefront reconstruction are given in [13].

The principle of Hartmann measurements is based on ray propagation: it does not depend on the wavelength and works with coherent as well as non-coherent sources. These sensors are, therefore, achromatic and can be made compatible with different varieties of X-ray sources.

The first X-ray wavefront sensors (WFS) were based on the Shack–Hartmann approach [14], using a matrix of Bragg-Fresnel zone plates (FZP) [15] to measure EUV laser WF [16,17,18]. The first order of the FZP focused the X-rays reflected by individual FZP, producing an array of bright spots on the detector. The noise was a combination of the 0th order of the FZP (unfocused beam), the scattered light and the detector electronic noise. The signal to noise was high ensuring a good detection of the spot centroids. The main advantage of Shack–Hartmann systems over Hartmann sensors is an enhancement of the signal-to-noise ratio due to the use of lenses. This approach has not been pushed forward because of the drawback of chromaticity of FZP. Since the focal length varies with the wavelength and since the detector has to be located at the focal plane, the detector has to be moved every time the wavelength is changed. Furthermore, with a Shack–Hartmann based on FZP, a narrow bandwidth source is required, restraining the use of such sensors. Therefore, EUV to X-ray Hartmann WFS were developed instead [9].

### 2.2. Design of A Hartmann Wavefront Sensor

The EUV to X-ray Hartmann WFS presented here are the results of a long collaboration between SOLEIL, LOA and Imagine Optic. Since the beginning, the goal was to develop sensors able to measure the diffraction limited wavefront (λ/14 root mean square (RMS), with λ as the wavelength). In both the EUV and X-ray domains, in order to achieve accurate WF sensing, the WFS must have the ability to measure very small shifts of the Hartmann spots, with a typical accuracy of 1/100th of a pixel. It is, therefore, mandatory to have an optimal design, combined to a very accurate data treatment.

When designing a Hartmann sensor, key parameters must be considered: (1) The holes should be small enough to create diffraction on the detector. (2) Crosstalk (interferences between neighboring spots) must be minimized, as they may induce deformation of the shape of the spots resulting in an error of the measured centroids. Therefore, to avoid crosstalk, the hole pitch should be large enough when working with partially or fully coherent beams. (3) The best spatial sampling is obtained when the Hartmann plate has holes as dense as possible. (4) To increase the WF sensor sensitivity, one may increase the distance from the mask to the detector. Considering all these requirements, the best trade-off for each targeted energy range and accuracy should be found in a case-by-case basis.

**Figure 2:**
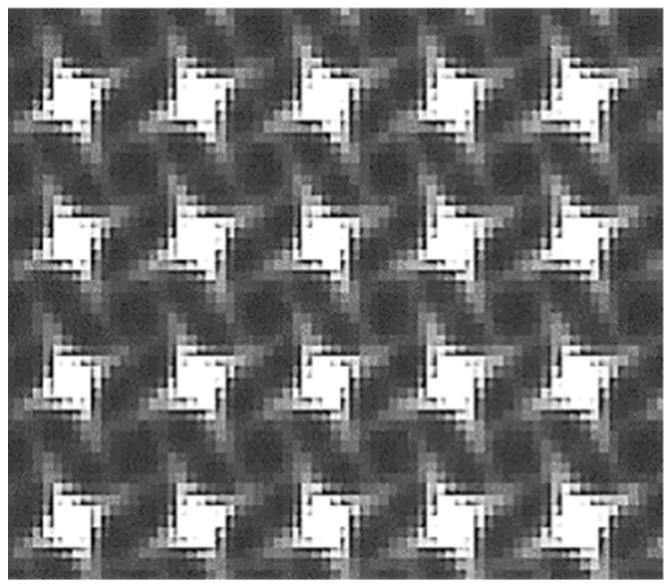
Raw image illustrating the diffraction pattern created by the grid of rotated square holes.

To answer above-mentioned points (2) and (3), Imagine Optic patented the use of rotated square holes [19]. Contrary to circular holes that have omnidirectional diffraction, the square holes create four-direction diffraction patterns, as illustrated in Figure 2. This design minimizes crosstalk, and thus, enables to increase the hole density.

At low X-ray energies (high wavelengths), crosstalk is the dominant source of errors, as the effect of diffraction is stronger. At high X-ray energies, where diffraction is weaker, the error in the Hartmann sensor is mainly dominated by the detector shot noise. The intensity variation might have an important impact on the centroid calculation when the diffraction is weak.

**Figure 3:**
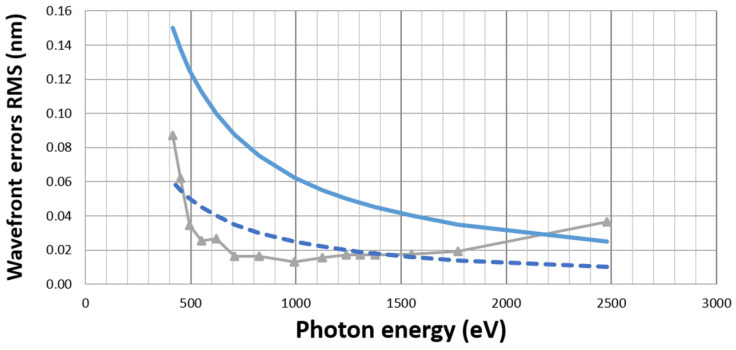
Simulation of wavefront errors (line with triangles) as a function of photon energy for a soft X-ray wavefront sensor design. The blue line corresponds to a wavefront error of λ/20 RMS, while the dashed blue line corresponds to λ/50 RMS.

When considering all these design parameters, it is possible to simulate the corresponding effects using a dedicated propagation algorithm. Figure 3 presents an example of the result of such a simulation in the case of a sensor designed for the soft X-ray range. The holes are 17 µm, with a pitch of ~160 µm. The grey line with triangles corresponds to the result of the simulation, considering all the systematic errors. The blue line corresponds to a target of λ/20 RMS and the dashed blue line to a residual error of λ/50 RMS. With this design, errors are below λ/20 RMS from 0.4 to 2.1 keV. This plot illustrates the facts that errors are minimum over a wide range of energies, and increase at higher energies (where the diffraction is weak and intensity variations determine the spot position) and at lower energies (due to diffraction as interference between spots occurs).

This type of simulation helps approaching the best design of a Hartmann system. Other critical parameters are the overall quality of hardware components, such as the mask (opacity, reproducibility of the hole pattern) and detector (signal to noise, photon response non-uniformity, spatial sampling of spots). Moreover, mechanical errors, such as an imperfect plate (not regularly spaced holes, curved hole plate) or a misalignment, must be considered experimentally during the calibration of the WFS.

### 2.3. Calibration

To measure the wavefront accurately, it is mandatory not only to accurately determine the spot positions with respect to those measured with a perfect wavefront, but also to compensate for all systematic errors in the system: this is the main objective of the calibration step. Tiny mis-adjustments of the holes array, such as e.g., residual rotation or twist, or in the real size of the pixels may have a large impact in the calculated wavefront.

The referenced centroids are obtained from a well-known beam at a wavelength chosen inside the calibration range. The perfect wavefront can be either planar or spherical. With this perfect wave, the inter-distance between spots is directly proportional to the curvature of the WF. A planar wave with a perfect WF is extremely difficult to produce in the EUV and X-ray domain. Therefore, for the calibration step, a spherical wavefront is preferred. The first solution to get a perfect spherical WF is to consider a beamline where the source is small enough and far enough to be considered a point source. Another solution is to insert a small circular hole on the beam that will diffract a known spherical wave [20].

It is possible to calibrate a Hartmann wavefront sensor at a given wavelength and the sensor measurement accuracy is ensured for a broad wavelength range. The sensor may keep its calibration over many years. Without calibration, the sensor can detect a change of wavefront (differential mode), as will be illustrated with applications in X-ray imaging (Section 5).

### 2.4. Examples of Wavefront Sensors Developed by Imagine Optic

Hartmann WF sensors covering a large spectral range from EUV to hard X-ray are currently produced by Imagine Optic. Depending on the energy range, Hartmann WF sensors are based either on direct detection camera, or indirect detection that requires the conversion of X-ray to visible photons. In the EUV and soft X-ray ranges (0.02 to 5 keV), specialized, direct detection cameras are typically used. The sensor may be either placed under vacuum (for example close to a focal spot) or in air with the sensor attached to the vacuum chamber. Imagine Optic’s standard EUV WF sensor (λ = 4–40 nm) has an absolute accuracy of λ/50 RMS (relative accuracy of λ/100 RMS). More recently, an EUV sensor adapted to strongly convergent or divergent beams having a numerical aperture as high as 0.15 was developed. It operates in vacuum, and therefore, can be positioned close to the focal spot, and covers an energy range from 28 to 124 eV. It has been tested on harmonics in solid with preliminary results showing the strong link between the interaction regime and the WF [13,21]. In the hard X-ray range, a WFS based on indirect detection was tested, providing WF measurement over a broad 5–25 keV energy range (HASO HXR–Imagine Optic). A luminescent crystal converts X-rays into visible light and is conjugated to a visible camera using a relay optical system. This sensor has a field of view of 3 mm × 3 mm, a spatial sampling of 20 µm, and provides a WF measurement accuracy better than λ/ 10 RMS.

## 3. Beamline Alignment and Focus Optimization Using WFS

According to the Marechal criteria, a λ/14 RMS wavefront leads to a Strehl ratio of 80%. A perfect wavefront (λ/14 RMS or better) gives a “perfect” point spread function (PSF), while an aberrated beam gives a focal spot far from the diffraction limit. Wavefront information of a beamline leads to the prediction of the PSF, and consequently, the capability to optimize and control the focal spot.

**Figure 4:**
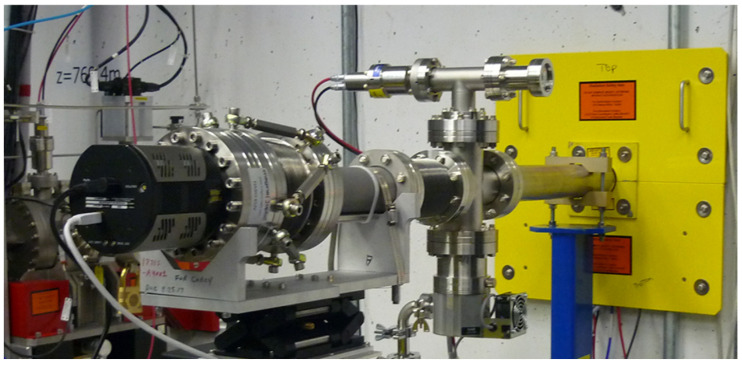
Soft X-ray wavefront sensor attached to the soft X-ray beamline at the Linac Coherent Light Source (LCLS). Reprinted with permission from [22].

Focusing optics, such as Kirkpatrick-Baez (KB) systems, can be optimally aligned to deliver the smallest focal spot. The first results presented were obtained at the AMO end-station of Linac Coherent Light Source (LCLS), at 1.3 keV (λ = 0.9 nm), thanks to a collaboration between Imagine Optic and Advanced Photon Source (APS), Lawrence Berkeley National Laboratory (LBNL), SLAC National Accelerator Laboratory and Brookhaven National Laboratory (BNL) under Department of Energy (DOE)/Basic Energy Sciences (BES) funding. A soft X-ray Hartmann WFS composed of 80 × 80 holes was used (Figure 4).

**Figure 5:**
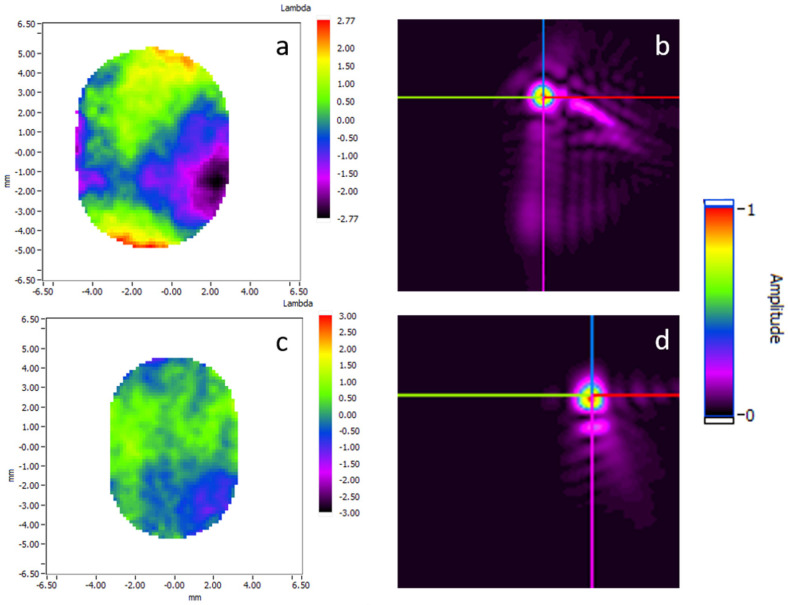
Wavefront maps before (**a**) and after (**c**) manual alignment of a Kirkpatrick-Baez. Energy distribution at the focal spot before (**b**) and after (**d**) alignment. Adapted with permission from [22].

With the KB at its initial position, the WF map was measured four different times, showing 1.12, 1.06, 1.08 and 1.02 nm RMS WF error. An example of the WF map is illustrated in Figure 5a, showing astigmatism at 0° as the dominant aberration. Application of back-propagation allowed to determine the energy distribution at the focal spot. Its profile, before the re-alignment, is illustrated Figure 5b and shows a 1.3 µm full width at half maximum (FWHM) spot size.

This KB system is based on bendable mirrors. In order to optimize the optical system, the influence functions of the KB were measured. The influence matrix was calculated and used to align the KB to reduce the number of aberrations. Figure 5c–d illustrate the results and show a residual WF error of 0.45 nm RMS (λ/2 RMS) as compared to the initial 1.06 nm RMS, and a reduced spot size FWHM down to 1µm. The typical shape of the coma was reduced by the KB re-alignment. A weak astigmatism is still visible, but much smaller than the initial one.

Such a wavefront monitoring alignment was performed at other wavelengths, for example at 32 nm at FERMI@Elettra [22,23,24] or at 10 keV at APS using the hard X-ray wavefront sensor (HASO HXR) [22], as well as with other kind of focalization optics: elliptic mirrors [25], Schwarzschild [13] or Wolter-like telescopes [26,27].

A WFS can also be used for automatic alignment of a beamline, as reported in [28]. The experiment was performed at the Swiss Light Source synchrotron (SLS) in collaboration with SOLEIL, LOA and Imagine Optic. The beam exiting the KB could be seen either by the Hartmann WFS, or, by a YAG: Ce crystal, positioned at the focal spot. The working energy was 3 keV (λ = 0.414 nm). A closed loop system was built between the KB, the WFS and the imaging system of the focal spot. The influence functions of the KB were measured, and the influence matrix was calculated and used to automatically align the KB. As a result, the level of aberration went from 7.7 nm RMS before adjustment of the KB down to 0.8 nm RMS after alignment, showing nearly 10 times improvement. The final WF appears mainly limited by shot noise. The focal spot went from 10.2 × 24.9 µm² to 6.8 × 10.2 µm² before and after re-alignment respectively and showed a much symmetrical shape.

**Figure 6:**
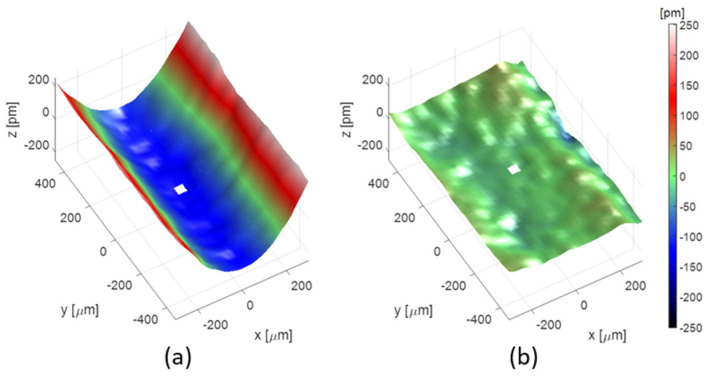
Wavefront map before (**a**) and after (**b**) the correction applied to the mirror.

A WFS can also be used to adjust in real time the shape of an active mirror to accurately drive its profile. The experiment was performed by Cocco and collaborators [29], based on a new focusing system called REAL, a water-cooled active optic mirror system. A close loop was installed to control in real time the beam WF. The experiment was performed at the Advanced Photon Source (APS), at 12 keV, using the HASO HXR WFS developed by Imagine Optic and Brookhaven National Laboratory (BNL). The first step consists of the measurement of the influence function of each heater. Before correction, the WF error was 114.5 pm RMS, while after automatic adjustment of the mirror, it decreased to 15.8 pm RMS, as illustrated by Figure 6. In this experiment, thermal bumps could be removed, and the surface quality of the mirror could be kept despite the heat load due to the incoming X-ray beam.

As illustrated in this section, a Hartmann WFS allows to probe the X-ray beam quality instantaneously and in situ. Coupled to an active mirror, automatic adjustments can be performed to optimize the wavefront and the focal spot.

## 4. Using WF Sensing to Both Understand the Physics and Optimize EUV Sources

Most UV and X-ray sources are impacted by stochastic processes, like for example amplification of spontaneous emission (ASE) for Free Electron Lasers or plasma-based EUV lasers, non-linear instabilities or gas turbulences for High Harmonic Generation. Seeded EUV or X-ray sources are intrinsically more stable, although stochastic processes may impact the coupling between the seed and the amplifier, such as the mechanical vibrations or remnant stochasticity of the seed. The ability to measure the build-up of spatial coherence and WF (together or independently) is an outstanding insight into the most fundamental processes related to the source creation. It gives a simple way to observe the linear and non-linear coupling between the wave and the emitting, and sometimes amplifying, medium.

Examples of the use of EUV WFS on many sources from High Harmonic Generation in gas, plasma-based EUV lasers (ASE and seeded) and Free Electron Lasers (self-amplified spontaneous emission (SASE) and seeded) will be presented in the following section.

### 4.1. Wavefront of High Order Harmonic Generation

High-order Harmonic Generation (HHG) created by the non-linear interaction of intense, often femtosecond laser with gas is becoming a reference source for numerous applications from atomic physic [30], magnetism [31,32], plasma physic [33], coherent imaging [34] and biology [35]. At the core of the HHG emission is the absorption of 2n + 1 infrared (IR) photons followed by the reemission of a single EUV or X-ray photon carrying the total energy of the absorbed photons and accumulating the information of the 2n + 1 incident photons. The spatial phase of a single harmonic is given by the summation of the electronic, atomic, the Gouy and the intensity-dependent dipole phases [36,37,38]. Thus, a tiny variation of the infrared laser WF, δφ_IR_ would impact the three-dimensional intensity variation around the focal spot, and leads to a variation of the HHG WF, δφ_HHG_ ≈ (2n + 1) δφ_IR_.

**Figure 7:**
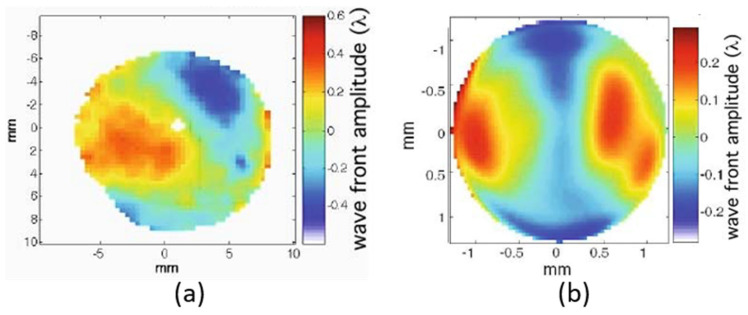
Wavefront maps in false color of (**a**) the High Harmonic Generation and (**b**) the infrared driving laser.

A typical WF obtained at 30 nm (27th harmonic) using Argon gas at 60 mbar is displayed in Figure 7a [39]. It is worth nothing that the HHG WF displayed are corrected from tilt and spherical aberrations that are dominant. Only the residual wavefront is measured, conceptually corresponding to the above-mentioned tiny shift from the focusing of infrared laser with a perfect WF. The driving laser was emitting at 800 nm at 1 kHz repetition-rate with energy per pulse of 1.2 to 1.4 mJ and 40 fs pulse duration. Each WF was averaged over 100 pulses. The RMS value was around λ_EUV_/5 RMS (Figure 7a), while the IR beam WF was measured at λ_IR_/9 RMS (Figure 7b). This result disagrees with the expected value, δφ_HHG_ ≈ 27 δφ_IR_ = 27 λ _IR_ /9 = 3 λ_EUV_ RMS for the HHG WF. It is interesting to observe that somehow the HHG WF copies the IR one, with astigmatism at 0° being the dominant aberration for both beams. This study was completed by more experiments [21,26,40].

**Figure 8:**
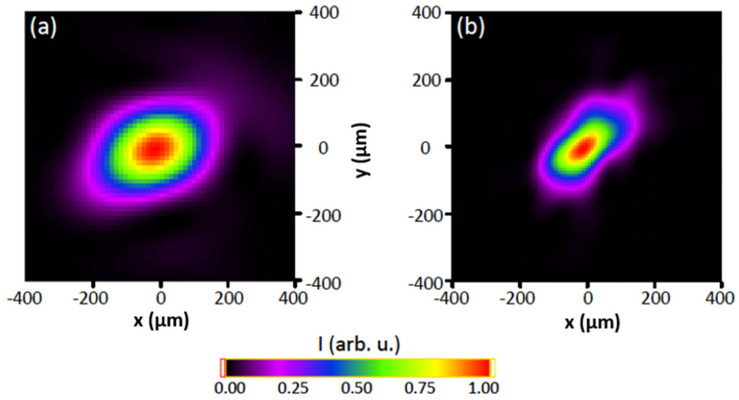
False color maps of the intensity distribution at the position of high harmonic emission for the infrared (**a**) and High Harmonic Generation (**b**) beams (adapted with permission from [26] © The Optical Society).

An extensive study was performed by the research group at Lund Laser Center, Sweden in collaboration with LOA [26]. Gas pressure, IR beam diameter, energy and spectral phase (Group Delay Dispersion) were varied to observe their impact on the WF. The driving laser WF was measured at λ_IR_/10 RMS, while the EUV WF ranged from λ_EUV_ /10 up to λ_EUV_ RMS in extreme cases, with λ ≈ 42 nm (19th harmonic). By using IR as well as EUV WFS, it was possible to calculate the intensity distribution of both beams on the plane of HHG (Figure 8). The IR spot and HHG source look very similar with a tilted elliptical shape. The HHG source is smaller, reaching nearly half the diameter of the IR spot along the smallest direction. This might be understood by the conversion efficiency, which depends non-linearly on the driving laser intensity. This also gives an explanation to the HHG WF, which was always measured to be much better than values given by simple calculation.

Several groups studied the harmonics generated by the combination of two laser pulses, the fundamental at 800 nm (ω) and its second harmonic at 400 nm (2ω) [41,42,43,44]. The simplest setup consists of inserting a thin (100 or 200 µm) Beta Barium Borate (BBO) crystal on the laser path after the focusing lens where the beam intensity is high enough to efficiently generate the second harmonic. Starting with a WF of λ_EUV_/7 RMS when using only the fundamental, the adjunction of the blue pulse yields to a strong improvement of the WF reaching λ_EUV_/17 RMS. In this specific collinear geometry, the 2ω pulse is more tightly focused than the fundamental pulse (by a factor of about 1.4). The region of strongest emission is, thus, reduced as compared to the case with the ω pulse only, leading to the spatial filtering of the incident laser [45].

All these experimental results show the close link between the IR laser wavefront and the resulting EUV beam wavefront.

### 4.2. Direct Optimization of High Order Harmonic Generation Wavefront

Knowing that the high harmonic WF is a kind of replica of the driving laser WF opened the exciting opportunity of controlling the high harmonic WF by adjusting the driving laser beam instead of using an EUV adaptive optics.

**Figure 9:**
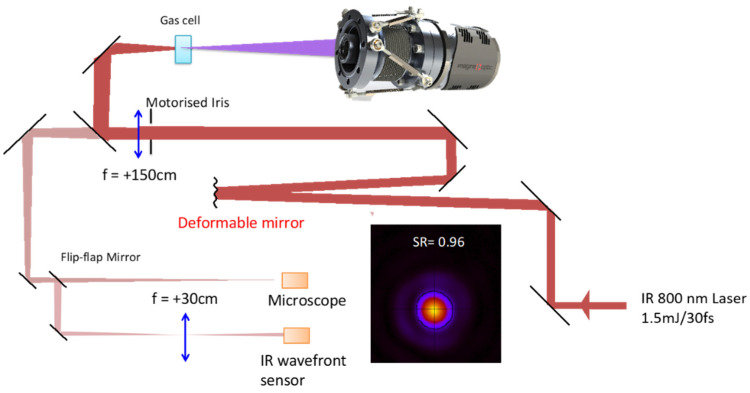
Schematic presentation of the experiment. The infrared laser path is displayed as the red beam while HHG is represented in purple. The picture shows an Extreme Ultra Violet (EUV) wavefront sensor manufactured by Imagine Optic. The image shows the best infrared focal spot achieved during the experiment and measured with the microscope.

For the first experiment [46], the IR WF was controlled by an Imagine Optic’s Shack–Hartmann sensor coupled with a deformable mirror composed of 31 actuators. Starting with a perturbed IR WF of λ_IR_/1.4 RMS with strong astigmatism, the HHG beam reached a WF of λ_EUV_/5 RMS. Then, decreasing the laser aberration down to its best of λ_IR_/7 RMS, the HHG WF did not change significantly reaching λ_EUV_/7 RMS. Although showing a kind of HHG control, several issues limited the effect of the active loop in this experiment. The system was, thus, optimized and tested again. The laser parameters were 4 kHz, 1.5 mJ, 35 fs, 800 nm. In the interaction region, Argon was used at a pressure of about 30 mbar. The MIRAO 52 deformable mirror from Imagine Optic has much smaller diameter (15 mm) than the previous deformable mirror (50 mm), leading to a full use of the actuators, even for the clipped beam. The IR focal spot was imaged with a microscope (Figure 9).

Before correction, the IR beam had a WF of λ_IR_/4 RMS that was improved to λ_IR_/30 RMS, leading to a Strehl ratio of 0.96. Then, the influence of different aberrations on the IR laser was qualified by observing the HHG beam cross-section measured 2 m away from the interaction region. Starting with a very slight astigmatism of 40 nm on the IR laser, the HHG beam was already elliptical, which is a clear signature of astigmatism. With no 0° astigmatism on the IR laser, the HHG beam was nearly circular. With +40 nm of 0° astigmatism, the HHG ellipse turned by 90°, proving the accurate control of the HHG astigmatism (results not shown).

**Figure 10:**
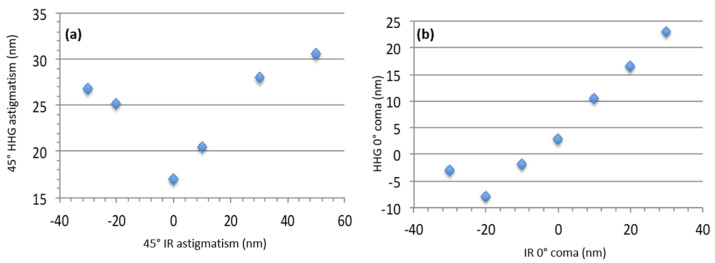
Variation of the HHG wavefront versus the IR driving laser wavefront considering pure 45° astigmatism (**a**) and pure 0° coma (**b**).

The HHG WF was measured and compared to the IR laser one for 45° astigmatism and 0° coma (Figure 10). By shaping the IR laser WF aberrations in, the HHG beam can be easily driven. However, it must be pointed out that the minimum 0° coma of the HHG beam was not found for the minimum of IR coma but for −20 nm. This seems to indicate that residual coma was introduced to the IR beam, probably by the focusing optics. The accuracy of the deformable mirror is probably still not enough to control very small aberrations in the high harmonic WF so as to reach the diffraction limit.

As a conclusion, the direct control of the HHG WF by modifying the IR laser one has been clearly established for 0°, 45° astigmatism and 0° coma.

### 4.3. Shaping High Order Harmonic Generation Wavefront

**Figure 11:**
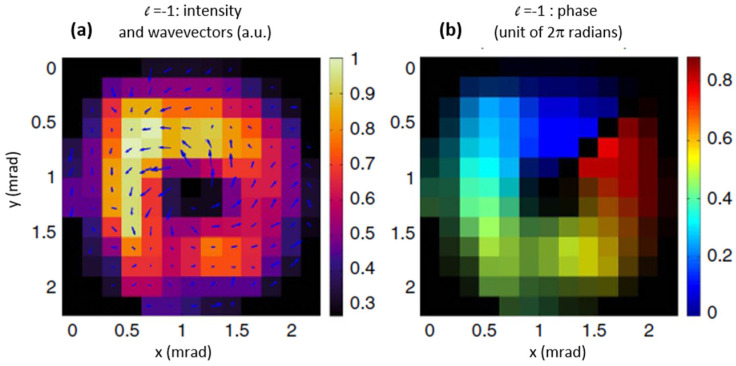
Intensity (**a**) and wavefront map (**b**) for the l = −1 mode of the 16th high-harmonic order emission (adapted from [50]).

Reduction of high harmonic aberration is the very first step in the active control of HHG WF. Today, several teams have succeeded to produce Laguerre-Gauss modes, also called vortices, in the EUV by placing an adequate phase plate on the IR laser path prior to the focusing lens [47,48,49]. The Hartmann WFS is ideal for measuring such rotating wave vectors that exhibits phase jump after a full rotation. An example is presented in Figure 11 [50]: the left figure corresponds to the intensity map with added wavevectors, clearly exhibiting a spiraling structure with increasing magnitude closer to the center of the beam. This is a signature of an optical vortex. The right one is the phase map.

A Hartmann wavefront sensor also allows to characterize the high charge vortex structure of the beams produced through HHG in a rare gas. This beam is generally composed of a set of numerous vortex modes. Their highly multimodal vortex structure was nicely described thanks to the EUV WFS [51,52].

### 4.4. Amplification of High Harmonic Beams; Seeded EUV Lasers

Apart from the High order Harmonic Generations, other EUV sources, namely Free Electron Lasers (FEL) and plasma-based EUV lasers (PBSXRL), produce collimated beams having laser-like characteristics. For a while, these sources were based on the amplification of the spontaneous emission, called SASE or ASE. Spontaneous emission is intrinsically stochastic, i.e., noisy. SASE and ASE sources are characterized by fluctuating parameters such as energy, divergence, pointing, spectrum for FEL only, coherence if any, and WF shape. This is detrimental for application experiments.

A high degree of spatial coherence appears spontaneously on FELs [53,54] and PBSXRL pumped by capillary discharge [17,18,55]. However, measurements achieved on SASE FEL showed strong fluctuations of the degree of spatial coherence due to the stochastic emission [56] and, thus, WF shape and quality [57,58]. The solution to improve the EUV beam quality consisted on seeding HHG beam inside FEL or plasma for amplification [59,60,61].

**Figure 12:**
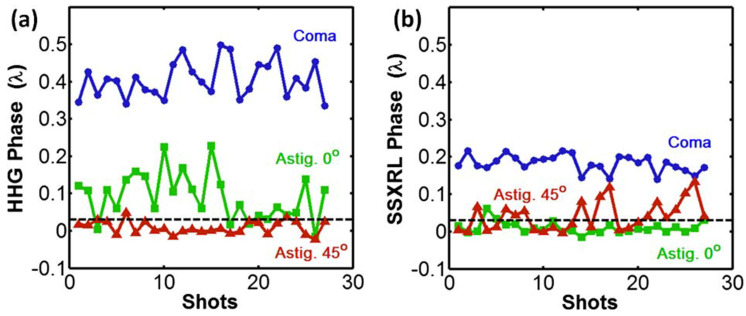
Shot-to-shot stability of the main aberrations for the High Harmonic Generation seed (**a**) and the seeded soft X-ray laser (**b**). Complementing the strong wavefront improvement, the wavefront is much more stable after amplification (reprint with permission from [21] © The Optical Society).

High harmonic seeding in plasma-based EUV laser has become a standard since 2004 [59] thanks to the impressive improvement of all the beam parameters: energy, divergence, polarization, coherence [62] and WF [63]. Here is presented an example of the most striking result relative to WF measurement: the WF of the HHG seed after amplification went from λ_EUV_/3 RMS (seed) to λ_EUV_/17 RMS (amplified beam), corresponding to Strehl ratios of 0.012 and 0.87, respectively. The initially elliptical beam having strong astigmatism is transformed into a diffraction-limited circular beam. This is a direct observation of the active spatial filtering achieved by the plasma amplifier [64]. With a WFS, an independent measurement of the level of the three main aberrations (coma, 0° astigmatism and 45° astigmatism) could be followed over time, in both the HHG (Figure 12a) and the seeded soft X-ray laser (SSXRL) (Figure 12b) beams. These plots illustrate that the plasma seeding is accompanied by an important improvement of the SSXRL stability [21]. The WFS provides information in real time of the stability of the beams and their aberrations.

## 5. Phase Imaging with Hartmann Wavefront Sensors

Hartmann WFS can also be used for imaging applications. We present here an example of plasma probing, and illustrate the importance to include the WF information to properly reconstruct an image in coherent diffraction imaging. Finally, we show that a Hartmann sensor can be used for non-destructive testing.

### 5.1. Plasma Probing

Mapping the electron density of laser-produced plasma is a major issue for the fields of inertial confinement fusion (ICF) and laboratory astrophysics. The electron density, N_c_, at which an incident light wave is reflected, is given by N_c_[cm^−3^] = 1.02 ∗ 10^21^ × λ^−2^ [µm]. In order to reach densities close to or above the solid density (~10^23^ e^−^cm^−3^), it is, thus, of prime interest to probe plasmas with EUV or X-ray beams. EUV Moiré [65] as well as EUV interferometry [66,67] have achieved 2D maps of electron density. However, these two techniques rely on complicated and expensive EUV optical systems [68]. Collimated, hard X-ray sources represent today the new paradigm in plasma imaging. Betatron is a laser-driven femtosecond source emitting photon of energies up to several 10’s of keV. This would ensure full penetration in today’s largest and densest laser-driven plasmas. However, this source is almost incoherent and emits a very broad spectrum, preventing the use of any interferometer or Moiré system.

**Figure 13:**
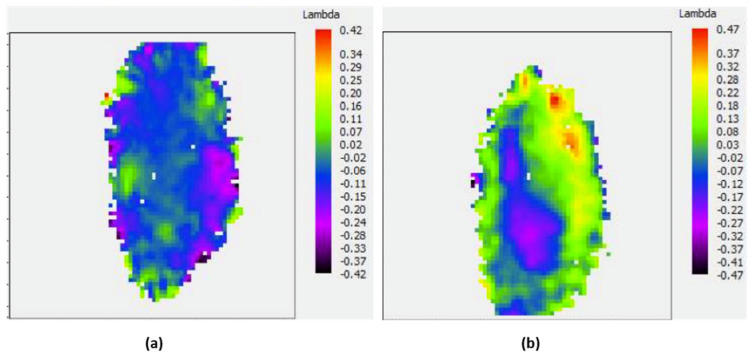
False color map of the 25th harmonic wavefront after passing through the 300 nm aluminum foil (**a**) before and (**b**) after the irradiation by the 3 keV FEL.

Plasma probing with a Hartmann sensor has been proposed long ago [69]. It regains interest with the advent of X-ray Free Electron Lasers [70]. A demonstration of the use of Hartmann WFS for plasma probing has been performed at LCLS Free Electron Laser. The plasma was created by the interaction of the XFEL pulse (3 keV, 35 fs pulse) with 300 nm aluminum foil [33]. The probe beam was the spectrally filtered 25th high harmonic generated from the interaction of 1 mJ Ti:Sa laser with Argon. Standard Imagine Optic’s EUV WFS was used to show the creation of the plasma by comparing the WF of the probe beam passing through the aluminum foil before and after the FEL interaction. The purple spot on the right image is due to the lensing effect of the plasma (Figure 13b). The beam is not well centered and, therefore, does not completely illuminate the sensor.

### 5.2. Improvement of Coherent Imaging by Use of Hartmann Wavefront Sensors

An image distortion may be caused by misalignment of optics. For coherent imaging, such as coherent diffraction imaging (CDI) or holography, the impact of the aberration is not observable in a straightforward manner. Data treatment is necessary to produce an image. If the object is not perfectly known a priori, which is the case for the majority of cases, it might be difficult to evaluate the image degradation. Studies have been performed to estimate and compensate for the effect of the curvature of the WF as generated for holographic microscopy in the visible range [71]. However, due to the availability of high-quality optics in the visible range, the question of the impact of any aberration on coherent imaging has been slightly investigated.

**Figure 14:**
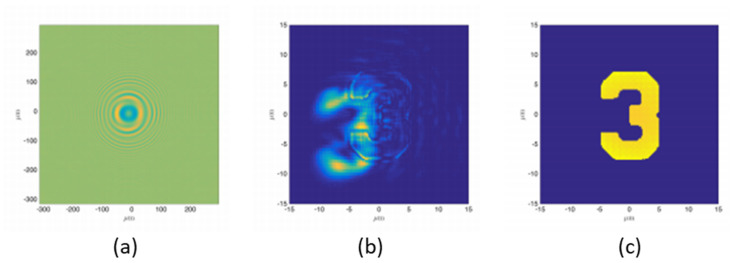
(**a**) Simulated hologram. (**b**) Reconstruction obtained using the general (planar waves) algorithm assuming no aberrations on the reference beam while in (**c**), the wavefront information was included in the reconstruction. X and Y axis are in µm. Adapted with permission from [72].

Collaborated research between IST, CEA and LOA has proposed a model to retrieve CDI images as if acquired using a beam with aberrations. The WF profile at the sample position was defined with the experimental Zernike coefficients, measured with a Hartmann WFS and an EUV HHG-based source focused by a Kirkpatrick-Baez optics system. Figure 14 shows the raw hologram (a), the retrieved image without considering the aberration in the incoming beam (b) and with correction from the HHG aberration (c). The impact of using the real HHG WF in the phase retrieval process impressively shows satisfactory reconstructions that can be achieved by including the aberrated phase map in the back-propagation [72].

### 5.3. Hartmann Wavefront Sensor Used for Non-Destructive Testing

**Figure 15:**
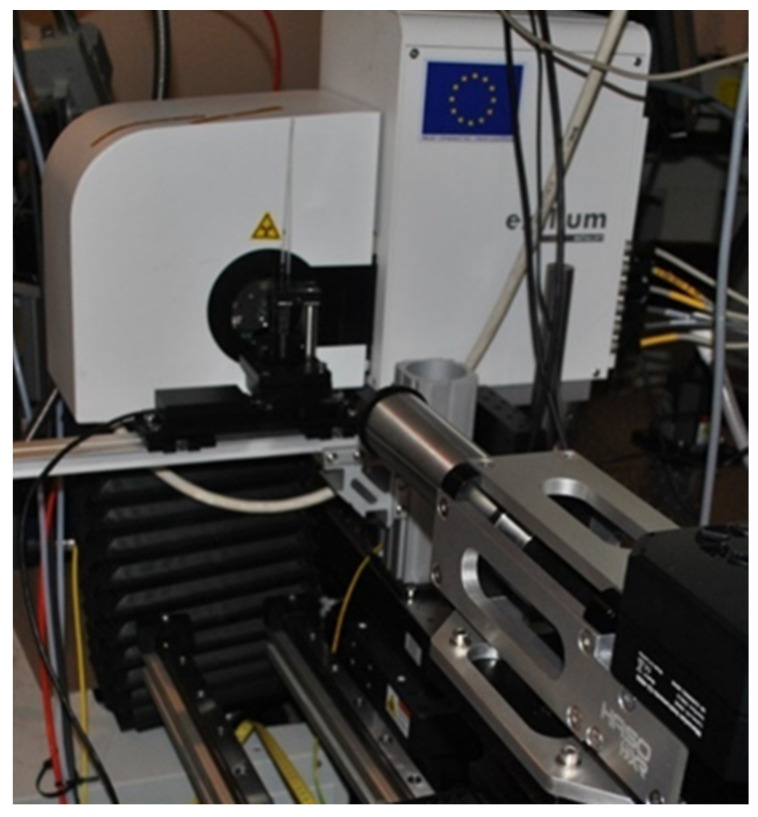
Experimental setup for phase imaging showing the Excillum X-ray source, the sample and the hard X-ray wavefront sensor (HASO HXR).

We describe here the use of a hard X-ray wavefront sensor from Imagine Optic (HASO HXR) to perform X-ray phase imaging [73]. The system is designed for use in a 5–25 keV energy range. It was placed in front an Excillum Metaljet microfocus X-ray source (D2 + 70 kV). A sample, composed of three tubes of 2 mm diameter (one PMMA tube, one polycarbonate capillary filled with water and one polycarbonate capillary full of oil), was positioned at 13 cm from the X-ray source and 27 cm from the sensor, leading to a magnification of 3.08 (Figure 15).

With this magnification, only a small part—one edge—of the sample is imaged. When the tube is filled with water or oil, X-rays have to go through two interfaces: between air and polycarbonate and between the polycarbonate and either the oil or the water. In the case of the PMMA tube, there is only one interface: air and PMMA. During the experiments, Hartmann images with and without the sample are systematically acquired. The intensity of each spot acquired with the sample was divided by the same spot without the sample (I/I_0_ map). Phase imaging was performed using relative WF measurements: the local deviations measured without sample were subtracted from the deviations of spots induced by the sample.

**Figure 16:**
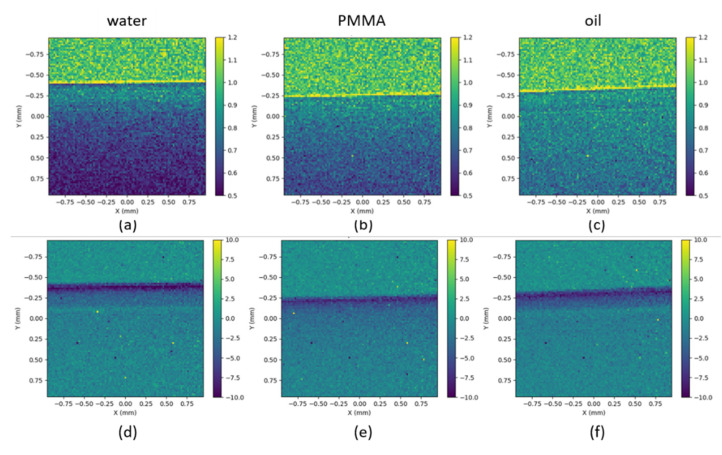
I/I_0_ map for the Hartmann mask for (**a**) the capillary with water, (**b**) the Poly(methyl methacrylate) (PMMA) and (**c**) the capillary with oil. An intensity ratio of one corresponds to the air (at the top of the three maps). Deflection (in µrad) maps for (**d**) the capillary with water, (**e**) the PMMA and (**f**) the capillary with oil. Sample magnification of 3.08. X and Y axis are in mm.

Figure 16 represents the I/I_0_ maps (a-b-c) and deflection maps (d-e-f) for the three samples. The transition from air (at the top of the images) to the tubes is clearly visible for all the samples on both transmission and deflection maps. The transition through the wall (50 µm) of the polycarbonate capillary is more visible in the deflection (d–f) than in the I/I_0_ (a–c) maps.

**Figure 17:**
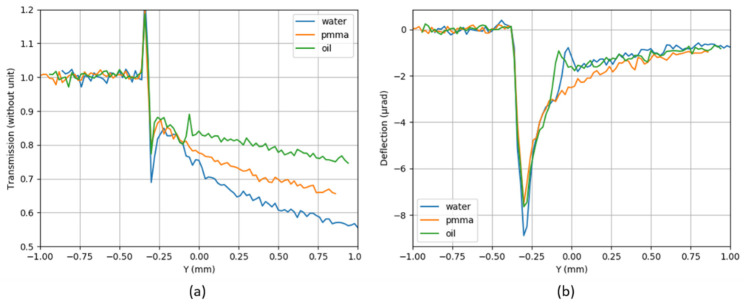
For the three samples (water in blue, PMMA in orange and oil in green): (**a**) average of the transmission map and (**b**) average of the deflection map along the Y direction.

The data presented Figure 16 were averaged over several lines and plotted along the Y direction in Figure 17. Refracted rays propagate towards the material which has a higher index of refraction. At the interface between polycarbonate (or PMMA) and air, the rays deviate towards the air; therefore, they have a negative direction along Y. This explains why there is a negative peak of ~8 µrad of deflection at Y = −0.3 mm for the three samples (Figure 17b). It corresponds to the interface air/material and indicates that the refractive index of the material (PMMA and polycarbonate) is smaller than the refractive index of air. Intensities from the refracted and non-deviated rays are added on the I/I_0_ map. The first peak located at −0.34 mm corresponds to the over-intensity resulting from the superposition of the non-deviated rays in air and the refracted rays coming from the edge of the samples (Figure 17a). It is typical of free-propagation results (as the deflections are measured on the plane of the mask, at a distance of 27 cm from the plane of the sample).

At the interface between polycarbonate and water (or oil), the rays are deviated towards the water (or oil) in a positive direction along Y. This explains why there is a relative positive deflection at the interface polycarbonate and water (or oil). The sign of the deflection is reversed, indicating that the refractive index of the material (oil/water) is larger than the one from the polycarbonate. The transition from polycarbonate to water could not be distinguished on the I/I_0_ plot, while it appears as a bump at Y ~ −0.02 mm in the deflection profile. The transition from polycarbonate to oil is slightly visible in the I/I_0_ plot, with a small maximum at Y = −0.06 mm. In the deflection profile, the transition is clearly seen at Y = −0.08 mm. For the oil, the two interfaces can be seen on both I/I_0_ and deflection profiles, while for water, it can be seen only on the deflection curve. There is a slight shift in positions due to the difference of indices between water and oil. This shows the high sensitivity of the sensor, and in the future, this may help to identify the constituents of an unknown sample. From −0.25 to 1 mm, deflection angles smoothly tend towards zero for PMMA.

With a smaller magnification, a larger size of the sample can be imaged. In this configuration, the transition between oil and polycarbonate could not be seen any more in the transmission profile due to insufficient spatial sampling.

**Figure 18:**
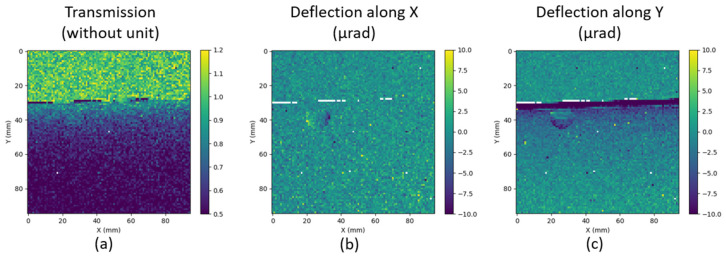
(**a**) Transmission map, (**b**) deflection map along the X direction and (**c**) deflection map along the Y direction obtained with the sample (polycarbonate tube filled with water) located at ~6 cm from the mask.

In a second configuration, the imaging system was at the same distance (40 cm from the X-ray source) and the sample was moved closer to the Hartmann mask, namely from 27 to 6 cm. The magnification is now 1.18. Figure 18 assembles I/I_0_ and deflection maps for the polycarbonate tube filled with water. With the current magnification, an air bubble can be clearly seen on the deflection maps, but this is not visible by absorption. Phase imaging allows to visualize structures that are not directly visible in absorption imaging. This capability is all the more important at higher energies, since materials tend to become homogeneous in terms of absorption with increasing energy.

## 6. Conclusions

This paper reviews the principle of a Hartmann wavefront sensor, the importance of calibration to perform absolute and accurate measurements of a wavefront, with an emphasis on the adaptation of the Hartmann approach to the EUV and X-ray domains. Knowing the wavefront of a beamline allows to optimize a focal spot by optimizing the alignment of the optics of a focalization system. Such sensors can also be used to better understand the physics of the X-ray sources, in particular for HHG or plasma-based sources or with table-top sources for non-destructive imaging.

As illustrated in this paper, Hartmann wavefront sensors have been used on many different facilities, such as FEL and synchrotrons, but also on High Harmonic Generation in gas and solid, as well as on laser-based EUV lasers.

As a conclusion, we summarize here the demonstrated benefits of Hartmann WF sensing in the EUV and X-ray range:

(1) With a calibrated system, from a single measurement (the raw image), the intensity, deflection and phase maps are obtained.
(2) The Hartmann approach is purely geometric and does not depend on the wavelength. The system is, therefore, achromatic. It can be designed to keep high sensitivity over a wide range of energy.
(3) The method is applicable to coherent, partially coherent as well as non-coherent radiation, which makes it appropriate for a wide range of X-ray sources (HHG, FEL, synchrotrons).
(4) Hartmann sensors are insensitive to mechanical vibrations during acquisitions, and thus, suitable for very long acquisition times.
(5) Hartmann systems are compact and easy to use and store with the sensor calibration data preserved.
